# Special Section Guest Editorial: Imaging Neuroimmune, Neuroglial, and Neurovascular Interfaces

**DOI:** 10.1117/1.NPh.9.3.031901

**Published:** 2022-09-30

**Authors:** Andy Y. Shih, Vanessa Coelho-Santos, Kıvılcım Kılıç

**Affiliations:** a Seattle Children’s Research Institute, Center for Developmental Biology and Regenerative Medicine, Seattle, Washington, United States; b University of Washington, Department of Pediatrics, Seattle, Washington, United States; c University of Washington, Department of Bioengineering, Seattle, Washington, United States; d Boston University, Department of Biomedical Engineering, Boston, Massachusetts, United States

## Abstract

The guest editorial provides an introduction to Parts 1 and 2 of the Neurophotonics Special Section on Imaging Neuroimmune, Neuroglial, and Neurovascular Interfaces.

In this Special Section of *Neurophotonics*, we focus on optical imaging techniques and their application to studies of neuroimmune, neuroglial, and neurovascular interfaces. Lacking its own energy reserve, the brain’s metabolic demands rely heavily upon a constant supply of blood. A dense network of microscopic blood vessels (arterioles, capillaries, and venules) ensures proper delivery of blood to the brain. However, the vasculature is not just static plumbing. It is highly reactive to surrounding neurons and its response can shape brain activity. In particular, the microvasculature can respond to vasoactive signals released by neighboring neurons and astroglia, and promote blood flow during periods of increased metabolic demand (i.e., neurovascular coupling).^1^ Many additional vascular functions are also crucial to brain health including regulation of molecular transport into and out of the brain via the blood-brain barrier,^2^ clearance of brain waste along cerebrospinal fluid (CSF)-filled perivascular routes,^3^^,^^4^ and regulation of immune cell entry into the brain.^5^ Our understanding of these vital functions has been accelerated by optical imaging techniques that facilitate both *in vivo* and *ex vivo* studies of the neurovasculature. The articles published here cover diverse topics and highlight a variety of innovative techniques and concepts from preclinical to clinical applications. They also showcase the exciting work being performed by young scientists that will usher in a new era of discovery.

The brain is composed of many functional regions with distinct circuitries. It is not surprising that the blood vessels serving these distinct tissues also differ in structure, cellular composition, cellular morphology, and genetic profiles.^6^^,^^7^ In addition to defining principles of blood supply to the brain, cross-regional comparisons will clarify why some brain areas are more vulnerable to pathology or why they generate different signals during fMRI. However, the scale of these cross-regional studies needs to be increased. Large-scale mapping of brain vasculature in fixed tissues is becoming more routine with simpler tissue clearing techniques and availability of light-sheet microscopy. Bennett and Kim discuss the various modalities to image whole brain vasculature, and weigh strengths and weaknesses of these approaches.^8^ They further discuss strategies to label different neurovascular cell types, which is crucial to understanding diversity in the neurovascular unit – the collection of cell types that comprise the blood vessel wall in different blood vessel types. In these endeavors, approaches are needed to label vascular cell types specifically and brightly.^8^ Frietas-Andrade et al. present an optimized approach to label the endothelium in fixed tissues and introduce software for analysis of vascular structure.^9^

When considering aging and age-related diseases, the cerebral white matter and deeper brain regions appear to be more vulnerable than cortex and the basis for this vulnerability has remained poorly understood.^10^^,^^11^ *In vivo* optical imaging has historically been limited to more superficial aspects of the cortex. Recently, three-photon imaging has become the tool of choice to image deeper into brain tissues at cellular resolution, allowing access to cerebral white matter, hippocampus and other subcortical structures without the need to remove overlying tissue.^12^^,^^13^ Thornton et al. describe how three-photon microscopy can be used for multi-color fluorescence imaging of white matter in mice using red fluorophores to image oligodendrocytes and neurons.^14^ This approach will be important for understanding the vascular basis of myelin degeneration in models of vascular cognitive impairment and Alzheimer’s disease.

While whole brain imaging is possible for fixed specimens, *in vivo* imaging remains more restrictive with small fields of view. There is a need to understand how behaviors of individual small arterioles and capillaries are coordinated to regulate blood flow broadly across vascular networks. To address this challenge, some imaging techniques strike a balance between imaging resolution, field of view, and acquisition speed. Optical coherence tomography angiography (OCTA) resolves microvascular structure and flow more broadly than multiphoton microscopy. Li and Tang describe the principles of OCTA and how to overcome challenges of tailing artifacts caused by blood vessels that limit 3D imaging capacity.^15^ Tournissac et al. describe a bihemispheric cranial window surgery to improve optical access for longitudinal imaging of brain vasculature *in vivo* at multiple scales and across brain hemispheres.^16^ Imaging broad fields of view is also critical to clinical applications. Miller et al. show how laser speckle contrast imaging can provide vascular flow information across a surgical field to guide neurosurgical procedures.^17^

How neurons communicate with blood vessels during neurovascular coupling is a topic of great interest and debate. Several signaling pathways may be involved. Astrocytes serve as an intermediary to convey signals from neurons to arterioles by enwrapping both synapses and the vascular wall.^18^ Cam H. Tran synthesizes current knowledge on astrocyte roles in neurovascular coupling and new imaging approaches.^19^ Gorza et al. provide a comprehensive review of opportunities and challenges to imaging subcellular astrocyte calcium activity using multiphoton microscopy.^20^ Recent work also suggests that neurons and astroglia provide signals to capillaries, which then rapidly conduct signals upstream to arterioles leading to vasodilation.^21^ Jeffrey et al. describe an isolated preparation with parenchymal arteriole and attached capillary networks ideal to probe the underpinnings of capillary-to-arteriole conductive signaling.^22^ Some experiments may require *ex vivo* brain slices approaches for rigorous pharmacology, and Bojovic et al. describe the use of acute brain slices as a model in neurovascular research.^23^ Studies are also increasingly in need of fast imaging of calcium and voltage sensitive dyes across tissue volumes; Har-Gil et al. provide new tools to address this need.^24^ This collection of approaches provides a strong platform for mechanistic studies.

Beyond neurons and astrocytes, other cells of the neurovascular unit are also being actively investigated. Vast capillary networks lie beyond the arterioles and blood flow regulation within capillaries is still not well understood. Recent studies have shown that capillary pericytes serve a role in local blood flow regulation but may contribute to slower aspects of this regulation.^25^ This may be due to distinctions in the contractile machinery of pericytes, compared to more dynamic tone changes controlled by smooth muscle cells. Erdener et al. provide a deep dive into the cytoskeletal elements that may control pericyte contraction.^26^ Occupying a perivascular niche next door to smooth muscle and pericytes of the arteriole-capillary transition zone, perivascular fibroblasts have recently come into the spotlight as potential regulators of vascular structure and function.^27^^,^^28^ They may regulate the structure of the vascular basement membrane, an acellular layer of matrix proteins in the vascular wall, or communicate with smooth muscle cells or pericytes. However, their physiological roles remain largely unknown. Jones et al. demonstrate how fibroblast localization can be mapped in cleared brains of fluorescent transgenic mice.^29^ Vascular cell types also communicate through release of extracellular vesicles, which carry signaling molecules and microRNAs with broad effects on brain function. Bağcı et al. describe approaches to define the molecular heterogeneity of extracellular vesicles.^30^

*In vivo* imagers are acutely aware of the observer effect, as one can easily disrupt the delicate physiological processes they intend to study. It is therefore paramount to use approaches that minimize the disruption of the brain and physiological state of the animal. Anesthetics commonly used during *in vivo* imaging shift our observations away from normal physiology. Zhang et al. provide a detailed guide on best practices to image and measure neurovascular responses in awake mice.^31^ Studying vascular development *in vivo* is also challenging because it is a highly orchestrated process^32^ easily derailed by neuroinflammation associated with skull removal during cranial window generation. Coelho-Santos et al. demonstrate how to minimize perturbations by creating thinned-skull windows in mouse pups for longitudinal imaging.^33^ Similarly, flow of cerebrospinal fluid (CSF) through perivascular routes during clearance of brain waste is a delicate process that can be disrupted by invasive cranial window implantation. Kiel et al. demonstrate how infrared imaging of CSF tracers can be used to track CSF tracer flux broadly in the brain through an intact skull.^34^

Immune cells within the blood, CSF, and marrow of the skull, can interact with and affect cerebrovascular function. This interplay becomes particularly important during brain disease, as recruited immune cells can release vasoactive substances, penetrate the brain tissue, obstruct blood flow, and damage vascular walls. Barkaway et al. provide a review that beautifully illustrates concepts and directions at this intersection between neuroimmunology and cerebrovascular research.^35^ Faulhaber et al. introduce easy-to-use reagents that label circulating immune cells for *in vivo* imaging that may facilitate new discoveries in this area.^36^ Further, Fukada et al. provide an innovative photothrombotic approach to occlude single brain vessels at depth that could be applied to precision studies of immune cell-vascular responses to brain injury.^37^

In summary, cerebrovascular research is clearly alive and well. There is no shortage of questions, powerful techniques, or innovative young researchers in our quest to uncover mechanisms of cerebrovascular function in health and disease. We are honored to have served as guest editors for this exciting collection of papers.

## References

[1] Girouard H. Iadecola C., “Neurovascular coupling in the normal brain and in hypertension, stroke, and Alzheimer disease,” J. Appl. Physiol. 100, 328–335 (2006). 10.1152/japplphysiol.00966.2005 16357086

[2] Zlokovic B. V., “The blood-brain barrier in health and chronic neurodegenerative disorders,” Neuron 57, 178–201 (2008). NERNET 0896-6273 10.1016/j.neuron.2008.01.003 18215617

[3] Iliff J. J. et al., “A paravascular pathway facilitates CSF flow through the brain parenchyma and the clearance of interstitial solutes, including amyloid β,” Sci. Transl. Med. 4, 147ra111 (2012). STMCBQ 1946-6234 10.1126/scitranslmed.3003748 PMC3551275 22896675

[4] Bakker E. N. et al., “Lymphatic clearance of the brain: perivascular, paravascular and significance for neurodegenerative diseases,” Cell. Mol. Neurobiol. 36, 181–194 (2016). CMNEDI 0272-4340 10.1007/s10571-015-0273-8 26993512 PMC4844641

[5] Marchetti L. Engelhardt B., “Immune cell trafficking across the blood-brain barrier in the absence and presence of neuroinflammation,” Vasc. Biol. 2, H1–H18 (2020). 10.1530/VB-19-0033 32923970 PMC7439848

[6] Shaw K. et al., “Neurovascular coupling and oxygenation are decreased in hippocampus compared to neocortex because of microvascular differences,” Nat. Commun. 12, 3190 (2021). NCAOBW 2041-1723 10.1038/s41467-021-23508-y 34045465 PMC8160329

[7] Pfau S. J. et al., “Vascular and perivascular cell profiling reveals the molecular and cellular bases of blood-brain barrier heterogeneity,” bioRxiv, 2021.2004.2026.441465 (2021).

[8] Bennett H. C. Kim Y., “Advances in studying whole mouse brain vasculature using high-resolution 3D light microscopy imaging,” Neurophotonics 9, 021902 (2022). 10.1117/1.NPh.9.2.021902 35402638 PMC8983067

[9] Freitas-Andrade M. et al., “Unbiased analysis of mouse brain endothelial networks from two- or three-dimensional fluorescence images,” Neurophotonics 9, 031916 (2022). 10.1117/1.NPh.9.3.031916 35620183 PMC9125696

[10] Meier-Ruge W. et al., “Age-related white matter atrophy in the human brain,” Ann. N. Y. Acad. Sci. 673, 260–269 (1992). ANYAA9 0077-8923 10.1111/j.1749-6632.1992.tb27462.x 1485724

[11] Reijmer Y. D. et al., “Improved sensitivity to cerebral white matter abnormalities in Alzheimer’s disease with spherical deconvolution based tractography, PLoS One 7, e44074 (2012). POLNCL 1932-6203 10.1371/journal.pone.0044074 22952880 PMC3432077

[12] Horton N. G. et al. “*In vivo* three-photon microscopy of subcortical structures within an intact mouse brain,” Nat. Photonics 7, 205–209 (2013). NPAHBY 1749-4885 10.1038/nphoton.2012.336 PMC3864872 24353743

[13] Ouzounov D. G. et al., “*In vivo* three-photon imaging of activity of GCaMP6-labeled neurons deep in intact mouse brain,” Nat. Methods 14, 388–390 (2017).1548-7091 10.1038/nmeth.4183 28218900 PMC6441362

[14] Thornton M. A. et al., “Characterization of red fluorescent reporters for dual-color *in vivo* three-photon microscopy,” Neurophotonics 9, 031912 (2022). 10.1117/1.NPh.9.3.031912 35496497 PMC9047442

[15] Li Y. Tang J., “Blood vessel tail artifacts suppression in optical coherence tomography angiography,” Neurophotonics 9, 021906 (2022). 10.1117/1.NPh.9.2.021906 35106321 PMC8785979

[16] Tournissac M. et al., “Cranial window for longitudinal and multimodal imaging of the whole mouse cortex,” Neurophotonics 9, 031921 (2022). 10.1117/1.NPh.9.3.031921 36159711 PMC9500537

[17] Miller D. R. et al., “Continuous blood flow visualization with laser speckle contrast imaging during neurovascular surgery” Neurophotonics 9, 021908 (2022). 10.1117/1.NPh.9.2.021908 35265733 PMC8900813

[18] Attwell D. et al., “Glial and neuronal control of brain blood flow,” Nature 468, 232–243 (2010). 10.1038/nature09613 21068832 PMC3206737

[19] Tran C. H. T., “Toolbox for studying neurovascular coupling *in vivo*, with a focus on vascular activity and calcium dynamics in astrocytes,” Neurophotonics 9, 021909 (2022). 10.1117/1.NPh.9.2.021909 35295714 PMC8920490

[20] Gorzo K. A. Gordon G. R., “Photonics tools begin to clarify astrocyte calcium transients,” Neurophotonics 9 021907 (2022). 10.1117/1.NPh.9.2.021907 35211642 PMC8857908

[21] Longden T. A. et al., “Capillary K+-sensing initiates retrograde hyperpolarization to increase local cerebral blood flow,” Nat. Neurosci. 20, 717–726 (2017). NANEFN 1097-6256 10.1038/nn.4533 28319610 PMC5404963

[22] Jeffrey D. A. Fontaine J. T. Dabertrand F., “Ex vivo capillary-parenchymal arteriole approach to study brain pericyte physiology,” Neurophotonics 9 031919 (2022). 10.1117/1.NPh.9.3.031919 PMC9225307 36278784

[23] Bojovic D. Stackhouse T. L. Mishra A., “Assaying activity-dependent arteriole and capillary responses in brain slices,” Neurophotonics 9, 031913 (2022). 10.1117/1.NPh.9.3.031913 35558646 PMC9089234

[24] Har-Gil H. et al., “Versatile software and hardware combo enabling photon counting acquisition and real-time display for multiplexing, 2D and continuous 3D two-photon imaging applications,” Neurophotonics 9, 031920 (2022). 10.1117/1.NPh.9.3.031920 36159710 PMC9487143

[25] Hartmann D. A. et al., “Brain capillary pericytes exert a substantial but slow influence on blood flow,” Nat. Neurosci. 24, 633–645 (2021). NANEFN 1097-6256 10.1038/s41593-020-00793-2 33603231 PMC8102366

[26] Erdener Ş.E. Küreli G. Dalkara T., “Contractile apparatus in CNS capillary pericytes,” Neurophotonics 9, 021904 (2022). 10.1117/1.NPh.9.2.021904 35106320 PMC8785978

[27] Dorrier C. E. et al., “Emerging roles for CNS fibroblasts in health, injury and disease,” Nat. Rev. Neurosci. 23, 23–34. NRNAAN 1471-003X 10.1038/s41583-021-00525-w PMC8527980 34671105

[28] Bonney S. K. et al. “Distinct features of brain perivascular fibroblasts and mural cells revealed by *in vivo* two-photon imaging,” J. Cereb. Blood Flow Metab. 42 (6) 966–978 (2021). 10.1177/0271678X211068528 34929105 PMC9125487

[29] Jones H. E. Abrams K. A. Siegenthaler J. A., “Techniques for visualizing fibroblast-vessel interactions in the developing and adult CNS,” Neurophotonics 9, 021911 (2022). 10.1117/1.NPh.9.2.021911 35402637 PMC8983066

[30] Bağcı C. et al., “Overview of extracellular vesicle characterization techniques and introduction to combined reflectance and fluorescence confocal microscopy to distinguish extracellular vesicle subpopulations,” Neurophotonics 9, 021903 (2022). 10.1117/1.NPh.9.2.021903 35386596 PMC8978261

[31] Zhang Q. et al., “Behavioral and physiological monitoring for awake neurovascular coupling experiments: a how-to guide,” Neurophotonics 9, 021905 (2022). 10.1117/1.NPh.9.2.021905 35639834 PMC8802326

[32] Coelho-Santos V. Shih A. Y., “Postnatal development of cerebrovascular structure and the neurogliovascular unit,” Wiley Interdiscip. Rev.: Dev. Biol. 9, e363 (2020). 10.1002/wdev.363 31576670 PMC7027551

[33] Coelho-Santos V. Tieu T. Shih A. Y., “Reinforced thinned-skull window for repeated imaging of the neonatal mouse brain,” Neurophotonics 9, 031918 (2022). 10.1117/1.NPh.9.3.031918 35673538 PMC9163199

[34] Keil S. A. et al., “Dynamic infrared imaging of cerebrospinal fluid tracer influx into the brain,” Neurophotonics 9, 031915 (2022). 10.1117/1.NPh.9.3.031915 35602461 PMC9113559

[35] Barkaway A. Attwell D. Korte N., “Immune-vascular mural cell interactions: consequences for immune cell trafficking, cerebral blood flow, and the blood-brain barrier,” Neurophotonics 9, 031914 (2022). 10.1117/1.NPh.9.3.031914 35581998 PMC9107322

[36] Faulhaber L. D. et al., “Antibody-based *in vivo* leukocyte label for two-photon brain imaging in mice,” Neurophotonics 9, 031917 (2022). 10.1117/1.NPh.9.3.031917 35637871 PMC9128835

[37] Fukuda M. et al., “Depth-targeted intracortical microstroke by two-photon photothrombosis in rodent brain,” Neurophotonics 9, 021910 (2022). 10.1117/1.NPh.9.2.021910 35311215 PMC8929553

